# Septal Reduction Therapy: Thinking Beyond the Conventional

**DOI:** 10.14740/cr2332

**Published:** 2026-10-24

**Authors:** Paulo Antonio Marra da Motta, Bernardo Drummond Motta, Carolina Gomes Cavalcanti de Oliveira, Fabricio Caied, Ernesto Misael Cintra Osterne, Thomas Edson Cintra Osterne, Evandro Cesar Vidal Osterne, Dinaldo Cavalcanti de Oliveira

**Affiliations:** a Department of Cardiology, Taguatinga Heart Institute (ICTCOR), Brasilia, DF, Brazil; b Department of Internal Medicine, Federal University of Pernambuco, Recife, PE, Brazil

**Keywords:** Obstructive hypertrophic cardiomyopathy, Alcohol septal ablation, Ethylene-vinyl alcohol copolymer, Ablation of marginal branch, Septal reduction therapy

## Abstract

For patients with obstructive hypertrophic cardiomyopathy who are refractory to medical treatment, the surgical myectomy is the treatment of choice, but for some patients, alcohol septal ablation (ASA) is an alternative. In the following case report, we described a patient that underwent ASA, but during the follow-up, the gradient came back. This patient did not have septal branches from left anterior descending artery; therefore, she underwent ablation of a marginal branch of the left circumflex artery using an ethylene-vinyl alcohol copolymer guided by evaluation of the invasive gradient (echocardiogram was not performed during the procedure). One year later, the patient presented with refractory symptoms, and color Doppler echocardiography revealed a mean resting left ventricle outflow tract (LVOT) gradient of 72 mm Hg, a basal septum thickness of 21 mm, and a mid- and apical-septal thickness of 10 mm. Coronary angiography showed a pattern similar to that observed at the end of the marginal branch occlusion procedure. A transcatheter treatment attempt was proposed to identify the vessel supplying the basal septum for potential ablation; however, the patient opted for surgical myomectomy, which was performed successfully and without complications. She was discharged on the fifth day of hospitalization. Based on this case, we conclude that septal ablation should not be performed without an echocardiographic assessment during the procedure, other branches than the septal branches may supply the interventricular septum (if ablation of these branches is performed, it must be as selective as possible), and the reduction of the invasive LVOT gradient should not be used as the sole criterion for deciding which branch to occlude.

## Introduction

Hypertrophic cardiomyopathy (HCM) is the most common genetic heart disease, and in its obstructive form, it involves the left ventricular outflow tract (LVOT). Invasive septal reduction is indicated for patients with a resting or provoked gradient LVOT ≥ 50 mm Hg who develop heart failure refractory to clinical treatment [1].

Myectomy is often not limited to excision of the interventricular septal muscle; it also addresses components of the mitral valve apparatus. It is the gold standard, if it is performed at a high-volume referral center with a mortality rate < 1% [2].

In the 1990s, three case reports were described in which alcohol was injected into septal branches of the left anterior descending artery, leading to a reduction in the LVOT gradient and improvement in symptoms. Since its description, alcohol septal ablation (ASA) has evolved in several aspects, and in cases in which myectomy cannot be performed, it is a therapeutic option for septal reduction therapy (SRT) [3].

Despite advances in ASA, there are several challenges with this technique, including the requirement for permanent implantation of a pacemaker, which ranges from 10% to 20% in some cohorts, and the inability to identify septal branches that are suitable for ablation [4, 5].

In the following case report, ablation of a marginal branch of the circumflex artery using a non-alcohol agent is described, as treatment for a patient with HCM and obstruction of the LVOT. One year later, the patient presented with refractory symptoms and a mean resting LVOT gradient of 72 mm Hg. We comment and discuss how to manage the case and what may be the reasons for the recurrence of the gradient.

## Case Report

### Investigations

A 61-year-old married female patient with HCM and significant obstruction of the LVOT, New York Heart Association (NYHA) class III heart failure refractory to clinical treatment, and no coronary artery disease according to coronary angiography, underwent successful ASA of the first septal branch in 2023, with no complications.

The patient underwent clinical and echocardiographic follow-up, but starting in the ninth month, she began to complain of dyspnea upon heavy and moderate exertion. The condition progressed to NYHA class III heart failure despite optimized medical therapy.

### Diagnosis

A color Doppler echocardiogram was performed, revealing a significant gradient in the LVOT. Coronary angiography showed small, slender septal branches arising from the left anterior descending artery, precluding any assessment of the gradient or the septal bulge (Fig. 1).

**Figure 1:**
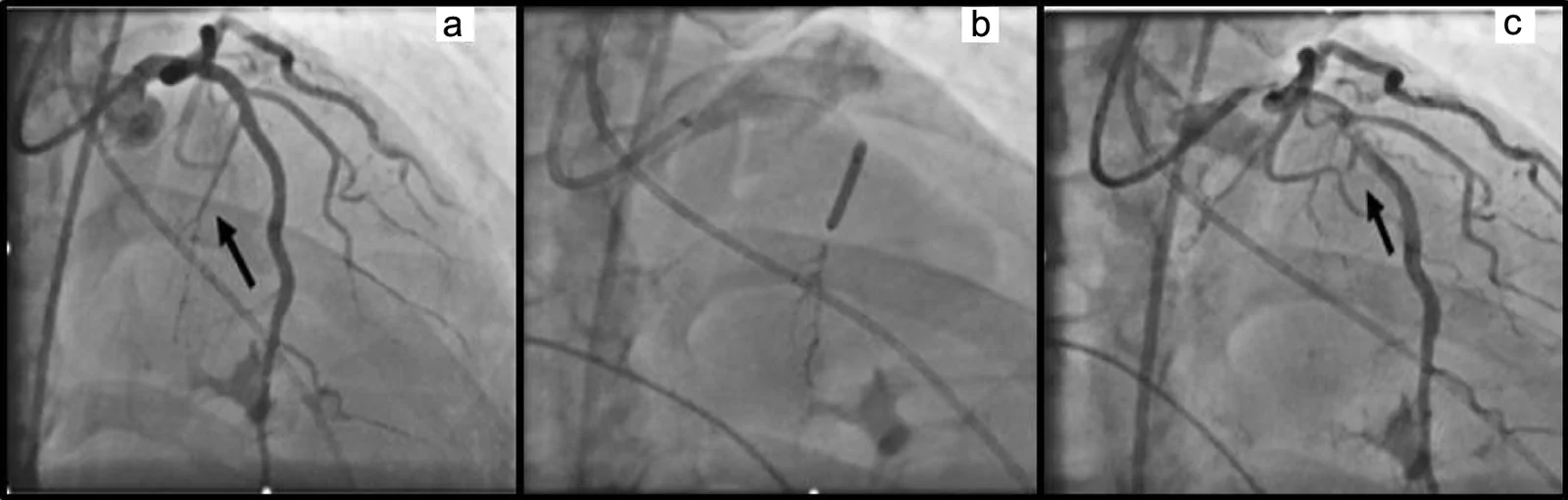
(a) Septal branch from left anterior descendent artery (arrow). (b) Alcohol septal ablation. (c) Occluded septal branch (arrow).

### Treatment

Given the patient’s angiographic status, the team performing the procedure conducted a balloon occlusion test in other vessels to simultaneously evaluate the LVOT gradient, without echocardiographic assessment of the perfusion area.

When the balloon catheter was inflated in the first marginal branch of the left circumflex artery (LCX), a significant drop in the gradient was observed.

After balloon withdrawal, a 0.014" guidewire was positioned in the marginal branch of the LCX. A microcatheter designed for use with ethylene-vinyl alcohol copolymer (EVOH) was placed distally in the marginal branch, and the 0.014" guidewire was removed from the coronary artery. An atraumatic neurological balloon was positioned and inflated in the LCX, occluding the origin of the marginal branch. The polymer was slowly injected through the microcatheter until it reached a point near the origin of the marginal branch; at that stage, the injection was stopped, the microcatheter was withdrawn, and the neurological balloon was deflated and removed from the LCX. Follow-up angiography demonstrated occlusion of the marginal branch and patency of the LCX (Fig. 2).

**Figure 2:**
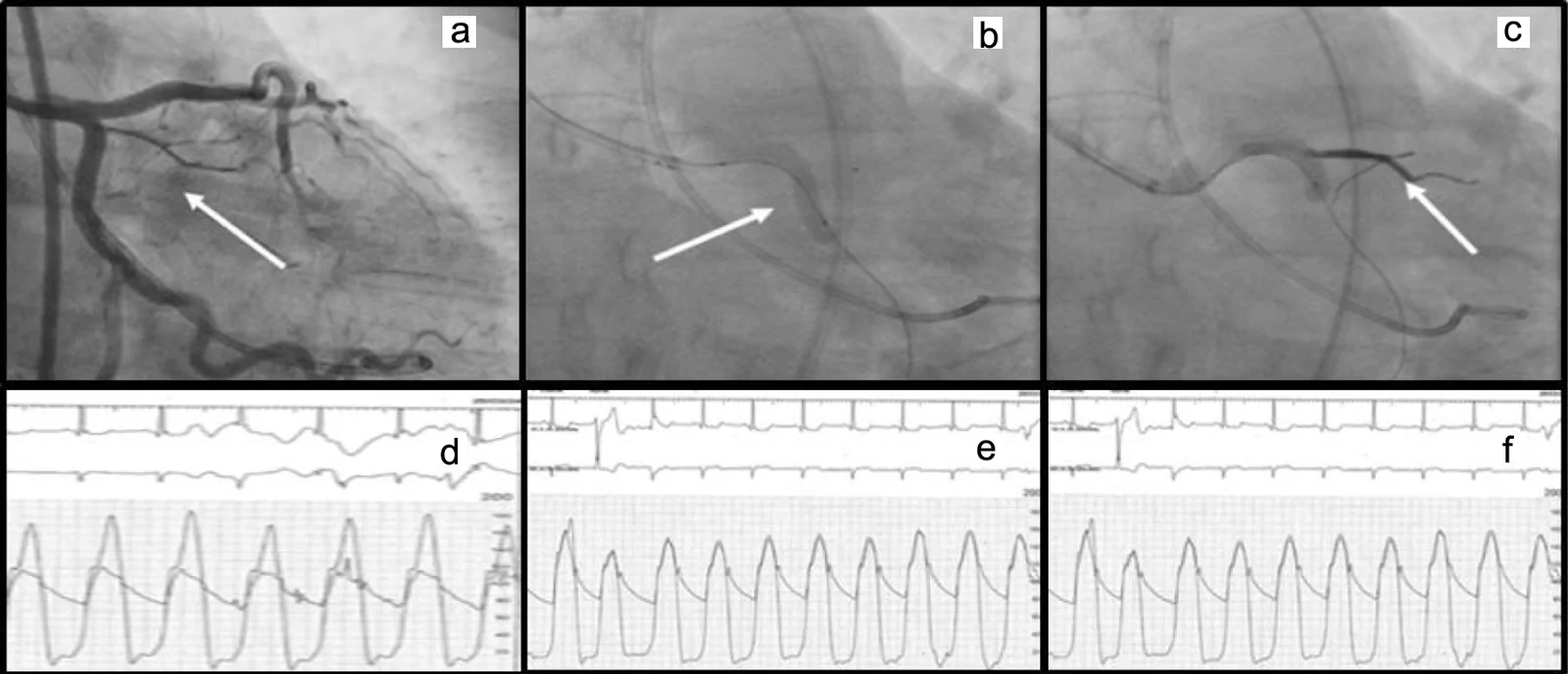
(a) Marginal branch from left circumflex artery (arrow). (b, c) Polymer posterolateral branch ablation (arrow). (d) Left ventricle gradient. (e, f) Reduction of the gradient after the procedure.

The patient was discharged on the fourth day; an echocardiogram (performed on the third day of hospitalization) revealed a mean LVOT gradient of 17 mm Hg, a basal septum thickness of 19 mm, and a mid-apical septum thickness of 12 mm.

### Follow-up and outcomes

One year later, the patient presented with refractory symptoms, and color Doppler echocardiography revealed a mean resting LVOT gradient of 72 mm Hg, a basal septum thickness of 21 mm, and a mid- and apical-septal thickness of 10 mm. Coronary angiography showed a pattern similar to that observed at the end of the marginal branch occlusion procedure (Fig. 3).

**Figure 3:**
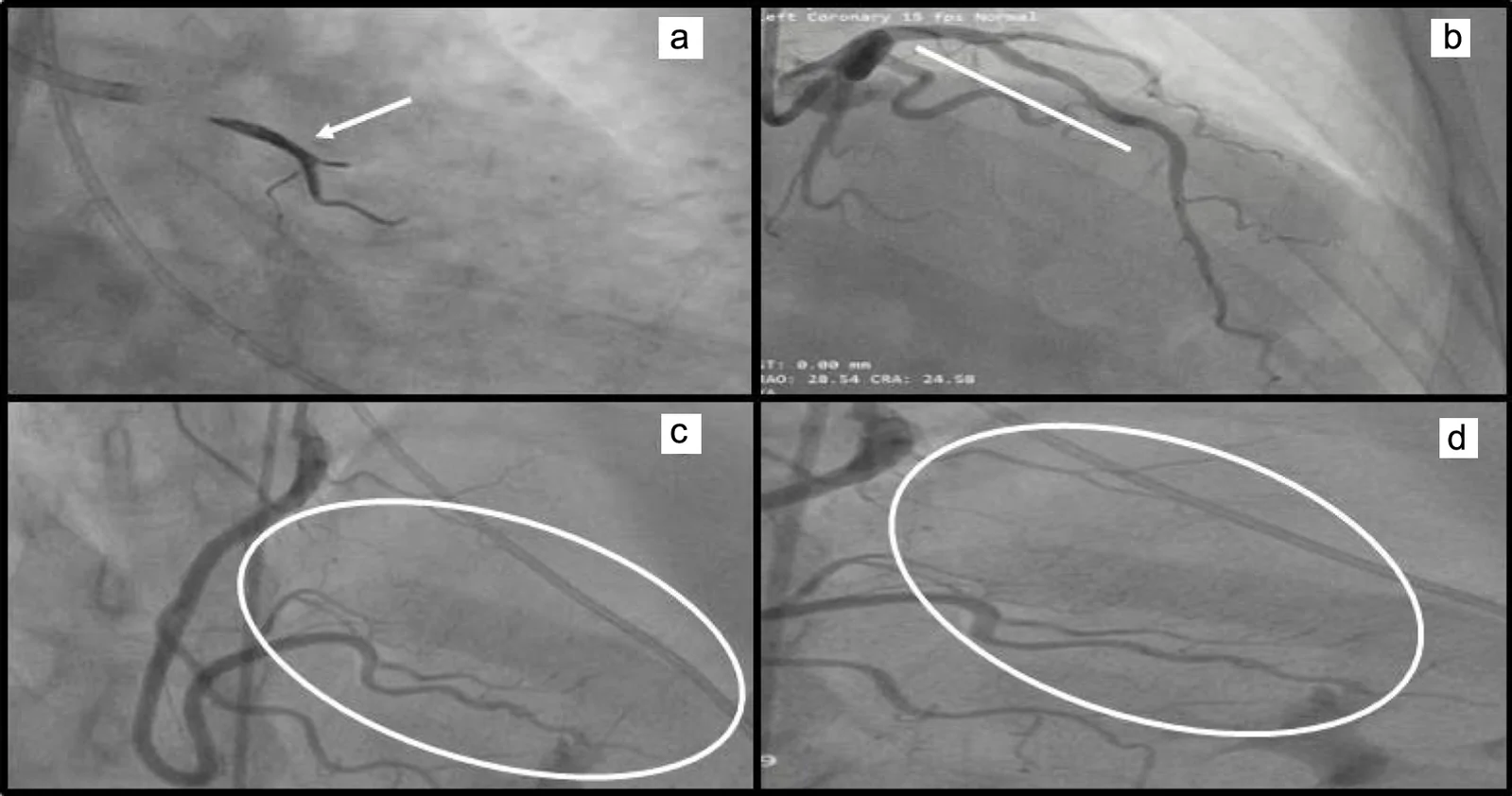
(a) Marginal branch occluded (arrow). (b) Left anterior descending artery without septal branches up to its mid portion. (c, d) Septal branches from right coronary artery.

A transcatheter treatment attempt was proposed to identify the vessel supplying the basal septum for potential ablation; however, the patient opted for surgical myomectomy, which was performed successfully and without complications. She was discharged on the fifth day of hospitalization.

## Discussion

ASA is established as an effective alternative to surgery as SRT for patients with HCM who have a resting or provoked LVOT gradient ≥ 50 mm Hg and NYHA class III or IV heart failure refractory to optimized clinical treatment [6, 7].

The Euro-ASA registry evaluated 1,275 patients who underwent ASA. It reported that the gradient decreased from 67 ± 36 to 16 ± 21 mm Hg and that the success rate was 89%. It should be noted that 30-day survival was 1%, 5-year survival was 89%, and 10-year survival was 77% [8].

A meta-analysis of 4,213 patients who underwent ASA, with a mean clinical follow-up of 6.6 years, revealed a pacemaker implantation rate of 10%, a post-procedural right bundle branch block rate of 60%, and a mortality of 1.5%/patient-year [9].

Since the 2000s, some authors have drawn attention to the fact that, in certain patients, the septal branches supplying the basal septum do not originate from the left anterior descending artery, but rather from the right coronary artery, diagonal branches, marginal branches, or other vessels. This anatomical variation is postulated to present in up to 15% of cases [10, 11].

It is important to emphasize that one of the major limitations of ASA is the inability to identify septal branches that are suitable for performing the procedure. This anatomical finding is present in 6% to 10% of candidates for transcatheter SRT [4, 5, 12].

A recurring question is how to perform SRT in patients without identifiable septal branches that are responsible for the LVOT gradient, given that the rationale for ASA is occlusion of a septal branch supplying the basal septum.

EVOH is an embolic polymer with an established indication for the treatment of cerebral arteriovenous malformations. EVOH is injected into the arteries through compatible microcatheters, which maintain its fluidity until it reaches the vascular bed [13].

Asil et al, Souza et al, and Martins Filho et al suggest that septal ablation with EVOH is efficient because the LVOT gradient was reduced to a single digit, there were no serious procedure-related complications, and the rate of the pacemaker implantation was less than 10% [14–16].

In the case report by Martins Filho et al [16], an LCX was occluded. Fibrotic burden in patients with HCM is a predictor of clinical events and death [6]. The medium- and long-term impact of LCX occlusion on a patient’s fibrotic burden is unknown, and we believe that selectivity regarding the amount of myocardium to be necrosed should be a target of SRT.

The distribution, morphology, and replication pattern of muscle sarcomeres, interstitial fibrosis, and function of the electrical conduction system in obstructive HCM follow a pattern that is specific to this disease. It is possible that characteristics that are specific to HCM are also associated with a distinct blood supply, and a distinct response to ischemia and necrosis [4, 2, 7, 12].

As previously stated, in some patients, no septal branches responsible for septal perfusion were identified, which precluded ASA [4]. However, it is clear that the blood supply to this region must originate from a coronary vessel; therefore, it is intuitive to expect that another artery may be responsible for perfusing this area.

In the present case, the marginal branch of the LCX supplied an area responsible for the LVOT gradient, and its occlusion resulted in a marked reduction in this gradient, but subsequent echocardiography demonstrated that the area of reduced motion was mid-distal rather than basal septal.

It should be noted that, given the vessel’s anatomical characteristics, embolization of the marginal branch using EVOH was performed. However, there was no echocardiographic assessment of the perfused muscle area during the procedure. Clinical follow-up revealed a recurrence of refractory symptoms and a resting LVOT gradient of 72 mm Hg.

The echocardiogram revealed a reduction in the mid-to-distal septum, but the basal septum was unaffected by the occlusion of the marginal branch. Coronary angiography of the right coronary artery reveals several very fine septal branches originating from the posterior ventricular (PV) branch and supplying the interventricular septum.

ASA of the aforementioned septal branches is not technically feasible; however, it would have been possible to occlude the mid-segment of the PV branch using an atraumatic balloon catheter and to inject echocardiographic contrast through a microcatheter positioned distal to the balloon to identify the perfusion area.

If this area were limited to the basal septum, occlusion of the mid and distal segments of the PV branch using EVOH could then be performed according to the described technique. However, the patient declined the transcatheter strategy and opted for surgical myomectomy, which was performed successfully and without complications.

Therefore, based on this case, we conclude that septal ablation should not be performed without an echocardiographic assessment during the procedure, other branches than the septal branches may supply the interventricular septum (if ablation of these branches is performed, it must be as selective as possible), and the reduction of the invasive LVOT gradient should not be used as the sole criterion for deciding which branch to occlude.

## Data Availability

The authors declare that data supporting the findings of this study are available within the article.
